# A hypothetical means of treating or preventing cancer

**DOI:** 10.1186/2049-3002-2-S1-O7

**Published:** 2014-05-28

**Authors:** Steven L McKnight

**Affiliations:** 1 Department of Biochemistry, UT Southwestern Medical Center, Dallas, TX 75390-9152, USA

## 

Mouse embryonic stem (ES) cells rely on threonine as a metabolic fuel. ES cells express the gene encoding threonine dehydrogenase (TDH) at a 1,000-fold higher level than any other mouse cell or tissue type. The mitochondrial TDH enzyme catalyzes the conversion of threonine into acetyl-CoA and glycine. The former metabolite feeds the TCA cycle, and the latter is consumed by the glycine cleavage enzyme complex to fuel one carbon metabolism. If compromised by nutritional, genetic or pharmacological means, inhibition of TDH kills mouse ES cells. By contrast, knockout mice lacking any TDH activity are viable, fertile and exhibit no phenotypic deficits.

Were TDH essential to the growth of any human tumor, one might predict that inhibitors of the enzyme would display favorable therapeutic utility. Unfortunately, among all mammals - including primates - humans are unique in being TDH-deficient. Proceeding with the idea that human tumors might exist in some other form of metabolic specialization, Benjamin Tu and I have followed an acetyl-CoA centric path in search of tumor-specific metabolic vulnerabilities. Numerous clinical studies using ^11^C-acetate PET imaging have reported tumor-enhanced uptake of acetate. Three enzymes are capable of converting acetate into acetyl-CoA; two of mitochondrial localization (ACSS1 and ACSS3), and a third that is nucleo-cytosolic (ACSS2). The McKnight and Tu labs, working collaboratively with the labs of Elizabeth Maher and Robert Bachoo, have found that the nucleo-cytosolic ACSS2 enzyme is primarily responsible for acetate uptake in cultured tumor cells and human tumors.

Knockout mice lacking ACSS2 are viable, fertile and devoid of obvious phenotypic deficits. When crossed with either of two genetic models of hepatocellular cancer, ACSS2-deficient mice exhibit a substantial reduction in tumor burden. Proceeding with these observations, a drug screen was performed in search of selective inhibitors of the ACSS2 enzyme. The screen yielded ACSS2 inhibitors that do not inhibit other acyl-synthetase enzymes, including ACSS1 and ACSS3. These inhibitors have been polished by medicinal chemistry to improve potency and pharmacological properties. As expected from the genetic ablation of ACSS2, potent ACSS2 inhibitors are well tolerated by mice and rats. Should it be the case that humans, like mice, are not reliant on the ACSS2 enzyme, it is possible that chemical inhibitor of ACSS2 may be of use in either the treatment or prevention of cancer.

